# Assessment of Socio-Economic and Climate Change Impacts on Water Resources in Four European Lagoon Catchments

**DOI:** 10.1007/s00267-019-01188-1

**Published:** 2019-11-08

**Authors:** Anastassi Stefanova, Cornelia Hesse, Valentina Krysanova, Martin Volk

**Affiliations:** 1 PIK—Potsdam Institute for Climate Impact Research, P.O. Box 601203, 14412 Potsdam, Germany; 2 UFZ—Helmholtz Centre for Environmental Research, Permoserstr. 15, 04318 Leipzig, Germany

**Keywords:** Eco-hydrological modeling, Socio-economic changes, Land use and water management, Climate change, European lagoons catchments

## Abstract

This study demonstrates the importance of considering potential land use and management changes in climate impact research. By taking into account possible trends of economic development and environmental awareness, we assess effects of global warming on water availability and quality in the catchments of four European lagoons: *Ria de Aveiro* (Portugal), *Mar Menor* (Spain), *Vistula Lagoon* (Poland and Russia), and *Tyligulskyi Liman* (Ukraine). Different setups of the process-based soil and water integrated model (SWIM), representing one reference and four socio-economic scenarios for each study area: the “business as usual”, “crisis”, “managed horizons”, and “set-aside” scenarios are driven by sets of 15 climate scenarios for a reference (1971–2000) and near future (2011–2040) scenario period. Modeling results suggest a large spatial variability of potential impacts across the study areas, due to differences in the projected precipitation trends and the current environmental and socio-economic conditions. While climate change may reduce water and nutrients input to the *Ria de Aveiro* and *Tyligulsyi Liman* and increase water inflow to the *Vistula Lagoon* the socio-economic scenarios and their implications may balance out or reverse these trends. In the intensely managed *Mar Menor* catchment, climate change has no notable direct impact on water resources, but changes in land use and water management may certainly aggravate the current environmental problems. The great heterogeneity among results does not allow formulating adaptation or mitigation measures at pan-European level, as initially intended by this study. It rather implies the need of a regional approach in coastal zone management.

## Introduction

While the use of climate scenarios in impact, adaptation, or vulnerability studies on water resources is well established, socio-economic scenarios are still given little attention. However, sound climate change impact assessments have to consider the implications of plausible socio-economic developments, since it is likely that societies will respond to climate change (Adger et al. 2003, 2005). Their responses will differ depending on the current socio-economic state and may considerably influence the environmental impacts of global warming. Or, seen from another perspective, climate change may intensify, weaken, or reverse certain environmental trends caused by human activities.

For these reasons, the next generation of climate change research should address the combination of climate and socio-economic scenarios to a larger extent than now. For instance, O’Neill et al. (2013) state that the four representative concentration pathways (RCPs) and the climate projections based on them should be related to shared socio-economic pathways (SSPs) representing plausible alternative trends in the evolution of society and ecosystems. Moreover, Kriegler et al. (2014) introduce the concept of shared climate policy assumptions (SPAs) representing climate change mitigation and adaptation measures. However, this new scenario matrix (RCPs–SSPs–SPAs) is not yet available for research questions, and no common guideline is yet available on how to implement societal changes in impact studies.

Several studies investigate land use change (LUC) in combination with climate change (e.g., van Roosmalen et al. 2009; Munoz-Arriola et al. 2009; D’Ágostino et al. 2010; Wang et al. 2014; Molina-Navarro et al. 2014; Morán-Tejeda et al. 2014; Gupta et al. 2015; El-Khoury et al. 2015), and a few papers also apply LUC projections based on socio-economic changes under climate change (Mehdi et al. 2015; Rouholahnejad Freund et al. 2017; Rajib and Merwade 2017), but to our knowledge there are no studies devoted to climate change considering impacts of land use and management changes.

In this paper, we contribute to closing this gap by assessing and intercomparing the impacts of future climate change under a set of different socio-economic scenarios in the catchments of four European lagoons. Lagoons and their drainage areas are complex ecosystems that are particularly vulnerable to climate change (Jeffress et al. 2012) and anthropogenic stress factors such as inappropriate exploitation of resources, intensive agricultural land use and management, and inappropriate water use and management (Anthony et al. 2009). Moreover, the hydrological regime and water quality of the inflowing rivers, which depend on the environmental conditions and overall ecological status of the catchment, can put additional pressure on the lagoon (Liu et al. 1997; Neil et al. 2002; Umgiesser et al. 2016; Vargas et al. 2017).

The main objective of our study is to provide a scientific basis for the development of an integrated water resources and coastal zone management strategy for European lagoons in the context of climate change. We analyzed a wide range of parameters regarding water availability and nutrients using an eco-hydrological model and a set of 15 climate scenarios in combination with four different socio-economic scenarios per case study area.

## Study Areas

**Fig. 1:**
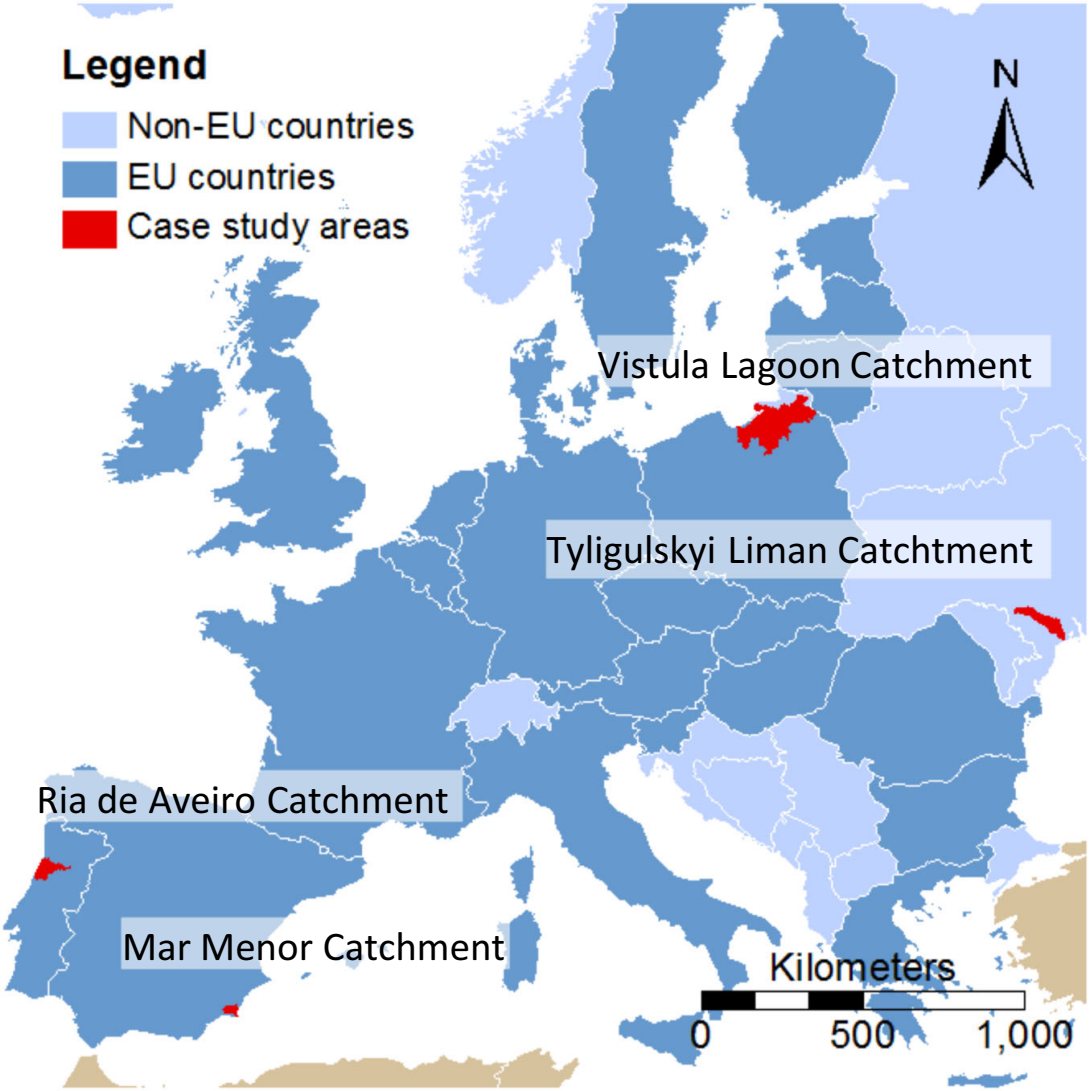
Locations of the four study areas

The study areas were selected to represent a diverse set of “hotspot” coastal areas in Europe (see Fig. 1). They are characterized by different environmental, climatic and socio-economic conditions (see Table 1) that come along with various pressures and future challenges. A SWOT (strengths, weaknesses, opportunities, and threats) analysis that was carried out prior to this study identified point source and diffuse pollution and inappropriate water management practices as the major environmental problems (Lillebø and Stålnacke 2015). Eutrophication is already an issue in all four lagoons and can be related to changes in the water quality and quantity of the inflowing rivers. The implications of climate change (e.g., lower runoff due to a decrease in precipitation) and of conflicting activities (e.g., intensification of agricultural activities at the cost of good water quality for recreation) are expected to put additional pressure on the lagoons in the future (Lillebø and Stålnacke 2015).

**Table 1:** Major characteristics of the four case study catchments

	Ria de Aveiro	Mar Menor	Tyligulskyi Liman	Vistula Lagoon
Inflow to lagoon [hm³ a^−1^]	2140	8.7	23	4035
Area [km²]	3556	1380	5240	20730
Altitude range [m a.s.l.]	−10 to 1105	−5 to 1061	−6 to 254	−27 to 308
Av. precipitation [mm a^−1^]	1100	337	515	750
Average temperature [°C]	14	25	10	8
Major River/s	Vouga	Albujon	Tyligul	Pregolya and Pasleka
Major land use (share in %)	Forest (56)	Agriculture (82)	Agriculture (80)	Agriculture (67)
Major soil type (share in %)	Cambisol (73)	Cambisol (76)	Chernozem (77)	Cambisol (38)

### Ria de Aveiro

The Ria de Aveiro is located in Northern Portugal and connected to the Atlantic Ocean. The catchment is largely covered by forest and hardly subject to environmental issues. However, the lagoon experiences high nutrient pollution originating mainly from point sources and agricultural areas located in the immediate vicinity of the water body (Hesse et al. 2013).

### Mar Menor

The Mar Menor is situated on the Mediterranean Coast in Southern Spain and has an intensely managed agricultural catchment. Since 1978, the area has received additional water for irrigation and public supply from the Tagus-Segura Inter Basin Water Transfer (IBWT) (Stefanova et al. 2015), which has led to drastic economic (e.g., horticulture under plastic and summer tourism) and environmental (e.g., jellyfish proliferation in the lagoon) changes over the last decades (Velasco et al. 2006; Lloret et al. 2005).

### Tyligulsyki Liman

The Tyligulskyi Liman lies on the Ukrainian Black Sea Coast. The catchment’s fertile soils (mostly Chernozems) are mainly used for agriculture, but agricultural production is far below its potential. Still, nutrient pollution is a serious threat for the Liman, as untreated urban waste waters are discharged into the rivers. Moreover, there are over 150 artificial fish farming ponds with a total volume of 19 × 10^6^ m³ (Tuchkovenko et al. 2012) that are refilled annually with water from the major rivers in the catchment, which causes a decrease of the freshwater inflow to the Liman by 35% (Tuchkovenko et al. 2015).

### Vistula Lagoon

The Vistula Lagoon has a transboundary catchment shared between Poland and the Russian exclave of Kaliningrad. Since the political changes in the year 1989, small farms that are frequently run by elderly people have dominated the agricultural sector in the Polish part, whereas on the Russian side only half of the arable land is cultivated compared with USSR times (Różyński et al. 2015). Hence, the agricultural sector has no significant impact on the environmental conditions in the catchment. Nutrient pollution is mainly an issue on the Russian side, where waste waters have been discharged practically untreated into the rivers for several decades. Since June 2017, an urban waste water treatment plant (UWWTP) that is located in the city of Kaliningrad has been collecting and treating most of the effluents. Changes in the hydrological regime of the catchment on both sides (Poland: diversion of the major inflowing river, the Vistula, and its branches to the Baltic Sea; Kaliningrad: transfer of 40% of the discharge of Pregolya river to the neighboring Couronian Lagoon) have led to a deepening of the water body due to more frequent wind wave re-suspension causing bottom erosion (Chubarenko and Margoński 2008), and to an increase of the salinity level of the lagoon (Kornijów 2018).

## Materials and Methods

### The Study Framework

**Fig. 2:**
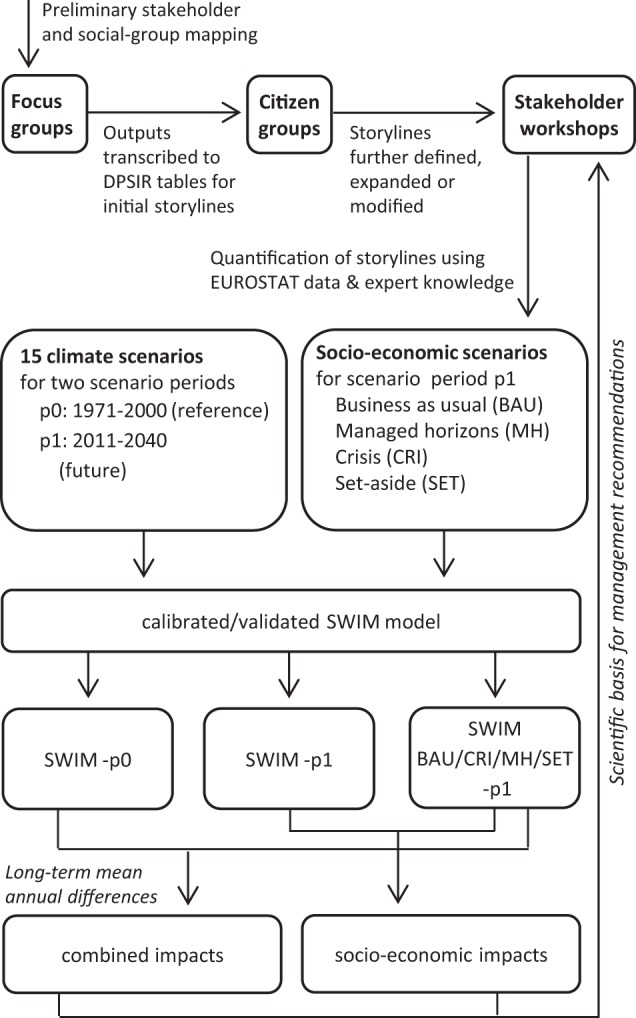
Flowchart of study framework. DPSIR: driving forces, pressures, states, impacts, responses framework developed by the European Environmental Agency to describe the interactions between society and the environment, EUROSTAT: the statistical office of the European Union, and SWIM: soil and water integrated model

In order to address future challenges related to climatic and socio-economic changes in the four catchments, a calibrated eco-hydrological model of each study area is driven by a set of downscaled climate data for a reference (1970–2011) and a near future (2011–2040) scenario period under different land use and management settings (socio-economic scenarios). The assessment is performed for socio-economic impacts only and combined impacts of socio-economic and climate change, which allows us to analyze the sensitivity and vulnerability of water quantity and quality indicators toward climate change and anthropogenic interventions. An overview of the study framework is given in Fig. 2.

### The Eco-Hydrological Model Soil and Water Integrated Model (SWIM)

The SWIM (Krysanova et al. 2000) is a semi-distributed, process-based tool simulating hydrological processes, nutrient dynamics, crop yields, and erosion. It has been successfully applied to river basins of different sizes in Europe, Africa, Asia, and South America for various research questions (Krysanova et al. 2015a). The model is based on the models SWAT (Arnold et al. 1993) and MATSALU (Krysanova et al. 1989) and has a three-level disaggregation scheme: basin—subbasins—hydrotopes, with hydrotopes being sets of units with the same land use and soil type within one subbasin. Processes, such as water flows, vegetation growth, and nutrient cycling are first calculated for each hydrotope, and then aggregated at the subbasin level, from where lateral flows of water and nutrients are routed to the basin outlet.

The input data include:

1. spatial data, in particular a DEM, a land use map and a soil map with attributed parametrization,
2. time series data, in particular daily climate data (minimum, maximum, and average temperature, precipitation, air humidity, and solar radiation), and
3. management data, such as information on major crops with their planting and harvesting schedules, fertilization (*N* and *P*) and irrigation practices, other abstractions and water transfers, as well as water discharge and water quality indicators from at least one gauging station for comparison with the simulated outputs.

SWIM was setup for each study area individually considering specific characteristics of each study area. In the cases of the *Vistula Lagoon* and *Tyligulskyi Liman* the WATCH climate data (Weedon et al. 2011) was used to drive SWIM due to poor coverage in time and space of the available climate station data. An overview of all data used to setup and drive SWIM for each of the four study areas is presented in Krysanova et al. (2015b).

SWIM was calibrated against observed river discharge and nutrient loads for each study area. In some cases only biweekly or average monthly values instead of daily data were available for comparison. Subsequently, calibration parameters, such as the groundwater delay time or correction factors of the curve numbers, were transferred to the ungauged parts of the catchments based on geophysical and hydrological similarities (e.g., similar aquifer, soil types, and land use). A detailed description of the calibration and validation procedure for each of the four study areas can be found in a separate report (Hesse et al. 2013), as well as in Stefanova et al. (2014a) (*Ria de Aveiro*), Stefanova et al. (2015) (*Mar Menor*), and Hesse et al. (2014) (*Vistula Lagoon*).

Although the model calibration was challenging, satisfactory results were achieved for all study areas (see Table 2) in terms of Nash–Sutcliffe efficiency (NSE) and percent bias (PBIAS). The NSE is a nondimensional criterion describing the squared differences between the observed and simulated values (Nash and Sutcliffe 1970). It can vary from minus infinity to one (Nash and Sutcliffe 1970), with values above 0.6 considered as good, and above 0.8 as very good modeling results (Moriasi et al. 2015). The PBIAS shows the long-term relative differences of observed against simulated values and should be as near as possible to 0 for best modeling results. A PBIAS of ±10–15% is considered as satisfactory, while PBIAS ≤±3% means very good modeling results in hydrological applications (Moriasi et al. 2015). In the case of Mar Menor, the coefficient of determination *r*² was calculated instead of NSE due to scarce data.

**Table 2:** Observed (obs.) and simulated (SWIM) average daily discharge (Q [m³ s^−1^]) during calibration/validation and achieved criteria of fit (NSE and PBIAS) for the major rivers in the four study areas

Catchment	Q [m^3^ s^−1^]			NSE	PBIAS
Rivers	Obs.	SWIM	Period		
Ria de Aveiro					
Águeda	6.5	6.6	01/02–12/05	0.8	5.6
Cértima	4.6	5.0	01/02–12/05	0.8	−8.0
Vouga	22.8	22.6	01/02–12/05	0.8	0
**Mar Menor** (biweekly time step)				*r*²	
Albujon	5.5	5.5	10/02–02/04	0.7	0.1
**Tyligulskyi Liman** (monthly time step)					
Tyligul	0.6	0.6	01/84–12/88	0.9	0.4
**Vistula Lagoon**					
Pasleka	18.8	17.6	01/07–12/09	0.7	−12.9
Pregolya	80.0	80.5	01/83–12/96	0.7	−0.6

The small number of water quality observations in all study areas did not allow a calculation of the common statistical model performance criteria. In the case of *Ria de Aveiro* we used nutrient concentrations, while in the other three cases nutrient loads but no corresponding discharges were available. We achieved satisfactory results regarding water quality calibration and validation based on visual comparison and by comparing the long-term means (see Table 3). SWIM simulated adequately seasonal dynamics of nitrogen and phosphorus, as well as peaks and recessions that are typical for diffuse and point source pollution. Detailed results on water quality calibration and validation, including water temperature and dissolved oxygen concentrations can be found in Hesse et al. (2013).

**Table 3:** Long-term averages of the observed and simulated annual loads/concentrations for the four study areas

Catchment	NO_3_-N [mg l^-1^]			NH_4_-N [mg l^-1^]			PO_4_-P [mg l^-1^]		
River	Obs.	SWIM	Period	Obs.	SWIM	Period	Obs.	SWIM	Period
Ria de Aveiro									
Antua	4.58	5.26	01/03–12/09	0.64	0.64	01/02–12/09	0.40	0.74	01/02–12/07
Vouga	1.21	1.18	01/02–12/09	0.15	0.22	01/06–12/09	0.03	0.03	01/03–12/09

	NO_3_-N [t a^−1^]			NH_4_-N [t a^−1^]			PO_4_-P [t a^1^]		
	Obs.	SWIM	Period	Obs.	SWIM	Period	Obs.	SWIM	Period
Mar Menor									
Albujon	154	113	02/03–02/04	35	30	10/02–02/04	2.54	2.57	10/02–02/04
Tyligulskyi Liman									
Tyligul	4.57	4.53	01/01–12/07	2.52	2.41	01/01–12/07	1.94	1.94	01/01–12/07
Vistula Lagoon									
Sum of 9 rivers	4384	4540	01/80–12/09	3024	2235	01/80–12/09	462	424	01/80–12/09

### Climate Scenarios

It is recommended to use downscaled climate data for regional impact studies, as these have higher resolution compared with global circulation models (GCMs) (e.g., Giorgi et al. 2001; Christensen et al. 2007; Tebaldi and Knutti 2007; Teutschbein and Seibert 2010). Moreover, the use of multiple scenarios is recommended, as it provides a variety of methods and boundary conditions that allows assessing, at least partly, the range of uncertainties related to climate modeling. The climate scenarios used in this study are GCM–RCM projections (Table 4) from the ENSEMBLES project (van der Linden and Mitchell 2009). They have a 25-km grid resolution and are based on the SRES A1B emission scenario, which is intermediate regarding atmospheric CO_2_ projection (Nakicenovic et al. 2000). We applied scenarios from this generation because projections based on the downscaling models forced by GCMs from the Coupled Model Intercomparison Project Phase 5 (Taylor et al. 2012) were not available at the time the project started.

**Table 4:** Climate scenarios (s1–s15) used for impact assessment in the four study areas

	GCM	RCM	Institute	Country
s1	HadCM3Q3	RCA3	Swedish Meteorological and Hydrological Institute (SMHI)	Sweden
s2	HadCM3Q0	HadRM3Q0	Hadley Center for Climate Predictions and Research (HC)	Great Britain
s3	HadCM3Q3	HadRM3Q3	Hadley Center for Climate Predictions and Research (HC)	Great Britain
s4	HadCM3Q16	HadRM3Q16	Hadley Center for Climate Predictions and Research (HC)	Great Britain
s5	HadCM3Q16	RCA3	Community Climate Change Consortium for Ireland (4CI)	Northern Ireland
s6	HadCM3Q0	CLM	Swiss Federal Institute of Technology Zurich (ETHZ)	Switzerland
s7	ECHAM5-r3	RACMO2	Royal Netherlands Meteorological Institute (KNMI)	The Netherlands
s8	BCM	RCA3	Swedish Meteorological and Hydrological Institute (SMHI)	Sweden
s9	ECHAM5-r3	RCA3	Swedish Meteorological and Hydrological Institute (SMHI)	Sweden
s10	ECHAM5-r3	REMO	Max Planck Institute for Meteorology (MPI)	Germany
s11	ARPEGE	ALADIN RM5.1	National Center for Meteorological Research (CNRM)	France
s12	ARPEGE	HIRHAM5	Danish Meteorological Institute (DMI)	Denmark
s13	ECHAM5-r3	HIRHAM5	Danish Meteorological Institute (DMI)	Denmark
s14	BCM	HIRHAM5	Danish Meteorological Institute (DMI)	Denmark
s15	ECHAM5-r3	RegCM3	International Center for Theoretical Physics (ICTP)	Italy

The ability of the applied scenarios to simulate real climate in the four study areas is satisfactory with regard to the seasonal behavior of temperature and precipitation (Hesse et al. 2013). However, it can be rated as poor when the long-term average values of observed and simulated climate data are compared (Hesse et al. 2013). In three of the four catchments, the total annual precipitation is overestimated on average (*Ria de Aveiro* by 20%, *Tyligulskyi Liman* and *Vistula Lagoon* by 30% each), while it is underestimated for the fourth one (*Mar Menor* by 10%). The simulated mean daily temperatures are on average about 1 °C higher compared with observed data for the catchments of *Ria de Aveiro* and *Tyligulskyi Liman*, and about 0.3 °C lower than station data for the other two cases (Hesse et al. 2013).

**Fig. 3:**
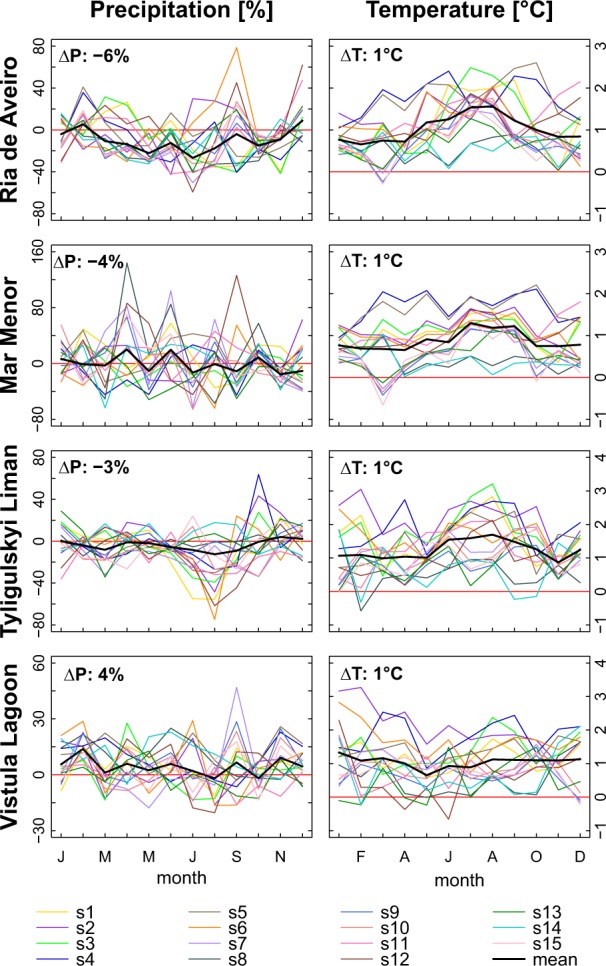
Long-term average monthly changes in precipitation and temperature for the scenario period (2011–2040) compared with the reference period (1971–2000) for the four study areas

Regarding future trends (2011–2040 compared with 1971–2000), temperature is projected to increase in all study areas by 0.9–1.2 °C on average, whereas there is no common trend for precipitation among the catchments (see Fig. 3 and Table 5).

**Table 5:** Long-term average annual climate change signals for temperature and precipitation in the scenario period (2011–20140) compared with the reference period (1971–2000) for the four study areas

	Ria de Aveiro			Mar Menor			Tyligulsyki Liman			Vistula Lagoon		
	P		T	P		T	P		T	P		T
	[mm]	[%]	[°C]	[mm]	[%]	[°C]	[mm]	[%]	[°C]	[mm]	[%]	[°C]
s1	−32	−3	1.2	19	8	1.1	−23	−4	1.5	25	3	1.2
s2	−74	−6	1.2	8	3	1.1	22	4	2.1	15	2	2.1
s3	−61	−4	1.2	−25	−6	1.1	−8	−2	1.8	9	1	1.4
s4	−60	−6	1.7	−18	−11	1.7	−11	−2	1.9	59	8	1.8
s5	−43	−4	1.7	1	1	1.6	−22	−5	1.5	91	11	1.4
s6	−82	−5	1.1	4	2	1.0	−8	−2	1.6	29	4	1.7
s7	−124	−7	0.8	−7	−3	0.6	−52	−11	1.1	21	3	0.8
s8	−282	−9	0.5	25	15	0.4	−25	−2	0.6	72	9	0.6
s9	−132	−9	0.7	−11	−5	0.5	−3	−1	0.8	56	6	0.6
s10	−191	−8	0.8	−28	−8	0.6	−45	−9	1.1	32	4	0.8
s11	−12	−1	1.3	8	4	1.1	−54	−11	1.2	0	0	0.8
s12	23	3	1.2	9	3	1.0	−43	−9	0.9	23	3	0.7
s13	−104	−4	0.7	−39	−11	0.5	11	2	0.8	−15	−2	0.5
s14	−975	−5	0.6	2	1	0.4	0	0	0.7	73	9	0.8
s15	−131	−7	0.7	−12	−3	0.5	−35	−5	0.9	25	3	0.8
Mean	−93	−6	1.0	−4	−2	0.9	−20	−3	1.2	34	4	1.1

In the case of *Ria de Aveiro* most scenarios project an overall decrease in precipitation, although many of them simulate considerably higher rainfall rates during the characteristic storms in fall. However, this is averaged out by a strong agreement among scenarios on a decrease in summer precipitation.

For the catchment of the *Mar Menor*, no clear average trend in precipitation can be identified. Almost half of the scenarios simulate an average annual decrease, but there is no consistency in seasonal changes which would allow further interpretation. Precipitation projections are also very diverse for the *Tyligulskyi Liman*. Nevertheless, a slight downward trend can be identified for average spring and late summer precipitation. In the catchment of the *Vistula Lagoon*, all scenarios except one project an overall increase in precipitation, however, there is no clear seasonal trend. The temperature increases, but does not follow a seasonal trend in this catchment.

### Socio-Economic Scenarios

We used four socio-economic scenarios per study area, each representing a combination of economic and environmental development for the near future. A detailed description of the scenario building process can be found in Baggett and Gooch (2015), while a general overview is given in Fig. 2. The basic ideas behind the scenarios can be shortly described as follows:

1. The business as usual scenario (BAU) represents a possible future, based on past trends. It assumes a positive trend in economic development with negative effects for the environment.
2. In the managed horizons scenario (MH), economic growth comes along with the introduction of appropriate measures that are considered beneficial for the environment.
3. The crisis scenario (CRI) represents a shrinking of the local economy paired with environmental degradation.
4. The set-aside scenario (SET) assumes a negative economic trend that has the potential to improve environmental conditions.

**Fig. 4:**
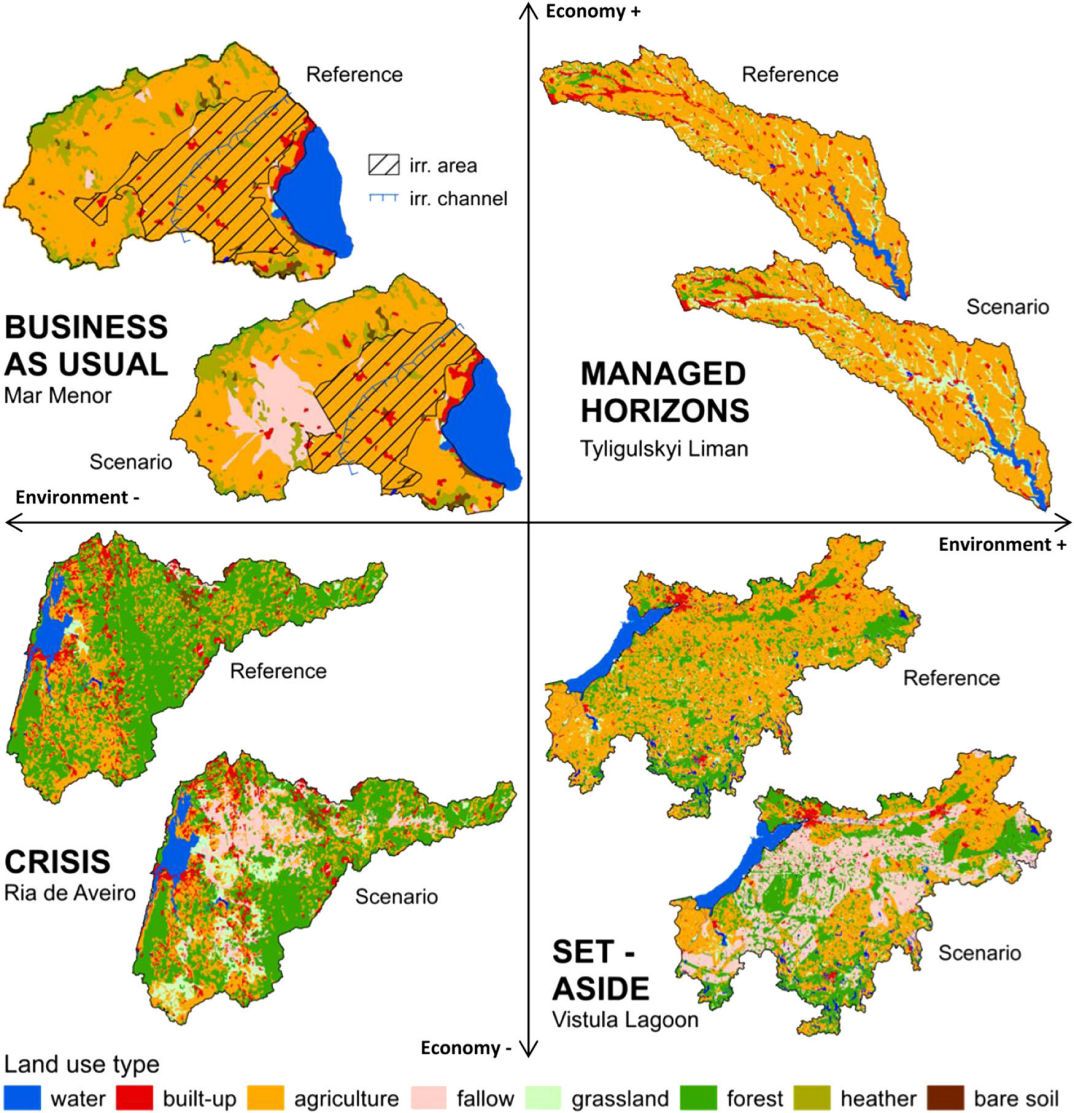
The reference land use maps and examples of scenario land use maps for the four study areas

By using the assumed relative changes in land use and applying a GIS tool new land use maps were created as input for SWIM. For that, soil quality criteria such as water holding capacity, terrain criteria such as morphology of the basin as well as the precipitation pattern were taken into consideration. Figure 4 shows examples of the scenario land use maps (one per study area). All scenario maps are presented in Stefanova et al. (2014b).

Changes in population size, tourism, and the level of sewage treatment were translated into changes in waste water discharges and nutrient loads from point sources (Table 6). Changes in agricultural practices implied solely changes in the use of mineral and organic fertilizers. In the specific case of *Mar Menor*, the irrigation zone of Campo de Cartagena was reduced/increased according to the assumed relative changes by excluding/adding areas furthest away from/closest to the main irrigation channel. Furthermore, in the catchment of the *Tyligulskyi Liman* the effective volume of existing ponds was reduced for the two scenarios seeking environmental benefits (MH and SET). In the two other cases no specific management changes were made.

**Table 6:** Assumed land use and management changes for the socio-economic scenarios (BAU, CRI, MH, and SET) in the four study areas

Changes in [%]	Ria de Aveiro				Mar Menor				Tyligulskyi Liman				Vistula Lagoon			
	BAU	CRI	MH	SET	BAU	CRI	MH	SET	BAU	CRI	MH	SET	BAU	CRI	MH	SET
Agriculture	−11	−16	–	−32	−14	−30	5	−15	–	–	−10	−30	1.4	−10	2	−50
Fallow	300	1200	−100	600	1500	3000	−100	1500	–	New	–	New	−9	New	–	New
Grassland	180	520	−100	−10	–	–	–	–	–	–	215	–	−6	–	−75	–
Forest	–	−26	3	5	–	−20	5	10	–	−50	10	40	−3	−20	17	86
Heather	–	–	–	–	–	–	−30	–	–	–	–	–	–	–	–	–
etlands	–	–	–	–	–	–	–	–	–	–	–	–	−5	–	−67	–
Point sources	−2	7	−8	−18	16	−10	5	−11	−8	−20	−50	−35	−10	−30	−40	−35
N_min_- and P- fertilizer	5	−20	−15	−20	–	−20	−15	−20	–	−50	500	200		−10	100	10
N_org_ - fertilizer	−10	−20	15	20	–	−20	15	20	–	10	10	−10	–	−10	300	–
Abstraction	6	−30	12	−15	–	–	–	–	−8	−30		−15	–	–	–	–
Discharge	–	–	–	–	22	−20	7	−11	–	–	–	–	–	–	–	–
Irrigation	–	–	–	–	−22	−45	5	−25	–	–	–	–	–	–	–	–
Ponds	–	–	–	–	–	–	–	–	–	–	−50	−75	–	–	–	–

## Results

In this section, first the water flows and major nutrient loads in the four study areas are presented under the reference conditions (1971–2000). Next, the effects of land use and management changes on discharge, major water cycle components, nutrient loads and nutrient fluxes for the near future scenario period (2011–2040) are evaluated.

### Discharge and Nutrient Loads under Reference Conditions

**Fig. 5:**
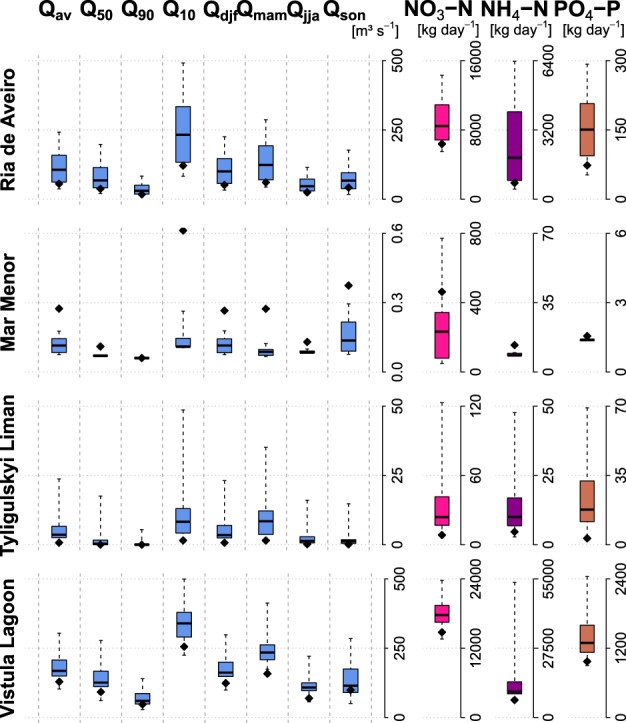
Average annual discharges and nutrient loads for the reference period using climate scenario data, shown as boxplots and observed climate data, shown as dots. The whiskers represent the min/max values, boxes the 25th/75th percentiles and thick lines the median values of SWIM outputs. On the left: Q_av_ (mean flow), Q_50_ (median flow), Q_90_ (low flow), Q_10_ (high flow), Q_djf_ (winter flow), Q_mam_ (spring flow), Q_jja_ (summer flow), and Q_son_ (autumn flow). On the right: NO_3_-N (nitrate nitrogen), NH_4_-N (ammonium nitrogen), and PO_4_-P (phosphate phosphorus) loads

Figure 5 shows the results of the simulated water and nutrient flow components for the reference scenario period (1971–2000) driven by climate models data and for the calibration periods driven by observed climate data. This comparison provides an indication of how well simulations under climate scenarios can reproduce the hydrological conditions in the catchment.

Water and nutrients flows simulated with scenario climate are on average notably higher than under the observed climate in the *Ria de Aveiro*, *Tyligulski Liman*, and *Vistula Lagoon* catchments, whereas they are lower in the *Mar Menor* catchment, which corresponds well to differences in precipitation shown in the scenario climate evaluation discussed in “Climate Scenarios”.

Discharge and nutrient inputs to the lagoons are quite diverse in terms of absolute values and climate sensitivity in the four study areas. High flow (Q_10_) shows the biggest range of variation among the analyzed variables in all study areas except *Mar Menor*, which indicates a high disagreement among climate models regarding the magnitude of storm events. Low flow (Q_90_) on the other hand shows the smallest variations, which does not implicate that it is less sensitive to climate, as we are looking at absolute values. The average annual discharge (Q_av_) is slightly higher than the discharge occurring during 50% of the time (Q_50_) in all four study areas, indicating higher significance of high flows than low flows for total flow. The ranges of the seasonal flows follow nearly the same regime as the corresponding discharges obtained during calibration, except for the *Tyligulskyi Liman* catchment, where these variations under the observed climate are hardly noticeable, probably due to water flow regulation.

The nutrient loads show a similar pattern like the average water flow components. They are slightly higher than the average values obtained under observed climate in the cases of *Ria de Aveiro*, *Tyligulskyi Liman*, and *Vistula Lagoon*, but lower in the catchment of *Mar Menor*. In this catchment the ammonium nitrogen and phosphate phosphorus loads to the lagoon show no climate influence, since they are mainly from point source pollution (UWWTP).

### Impacts on Water Availability

#### Changes in the average, median, high, and low flows

**Fig. 6:**
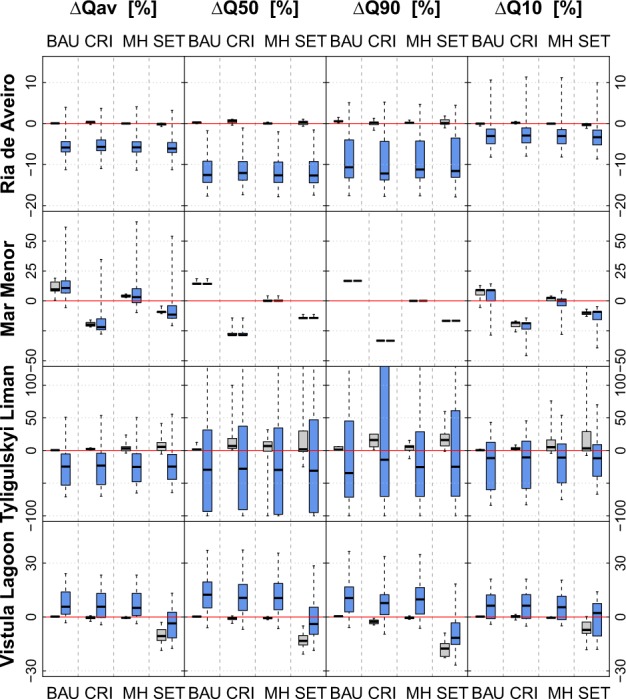
Average annual changes in discharge (Q_av_—mean discharge, Q_50_—median discharge, Q_90_—low flow, and Q_10_—high flow) for each socio-economic scenario (BAU, CRI, MH, and SET) with (in blue) and without (in gray) climate change shown as boxplots

Figure 6 presents the socio-economic and combined impacts on water flows in the four catchments. The assumed socio-economic changes hardly influence the analyzed variables in the *Ria de Aveiro* catchment and for three scenarios in the *Vistula Lagoon* catchments, whereas they strongly affect discharge in the *Mar Menor* and *Tyligulskyi Liman* catchments.

In the *Ria de Aveiro* catchment agricultural land is not dominant and water management plays a minor role, which explains the negligible effects of all four socio-economic scenarios. Adding climate change to these impacts a decrease in discharge can be observed. Relative changes and uncertainties among scenarios are largest for Q_50_ and Q_90_, both of which are very low under the reference climate. Hence, even small variations in climate have a relatively large impact on them.

The *Mar Menor* catchment is intensely managed and only about one third of the total inflow to the lagoon is generated naturally (Stefanova et al. 2015). Consequently, the combined impacts do not differ much from the socio-economic impacts. The assumed changes in released water from the UWWTP and the extent of irrigated agricultural area are well reflected by the variations in the four variables. Q_90_ and Q_50_ respond slightly more sensitively to these changes than Q_10_ and Q_av_, which is due to their dependency on infiltrated irrigation water and continuous input of water from the UWWTP. In contrast, changes in Q_10_ and Q_av_ are intensified or reversed some scenarios when climate change is added to the socio-economic impacts.

The socio-economic scenarios for the *Tyligulskyi Liman* catchment lead to a clear increase in discharge (e.g., 30% for Q_10_ in SET scenario), which is reversed by climate change (−20% on average). Moreover, the combined impacts on Q_90_ and Q_50_ show considerable uncertainty reaching values of up to +500% in some scenarios. These big relative changes are the result of extremely low absolute values during the reference period, and a heightened sensitivity of the variables to climate change.

In the *Vistula Lagoon* case, a strong reduction of agricultural land (−50%) and its conversion to fallow and forest in the SET scenario lead to higher evapotranspiration rates and to a notable decrease in discharge. Climate change weakens this decrease, but the four variables still remain negative on average. With regard to the other three socio-economic scenarios, we observe an overall increase in discharge through the effect of climate change.

#### Changes in seasonal stream flow components

**Fig. 7:**
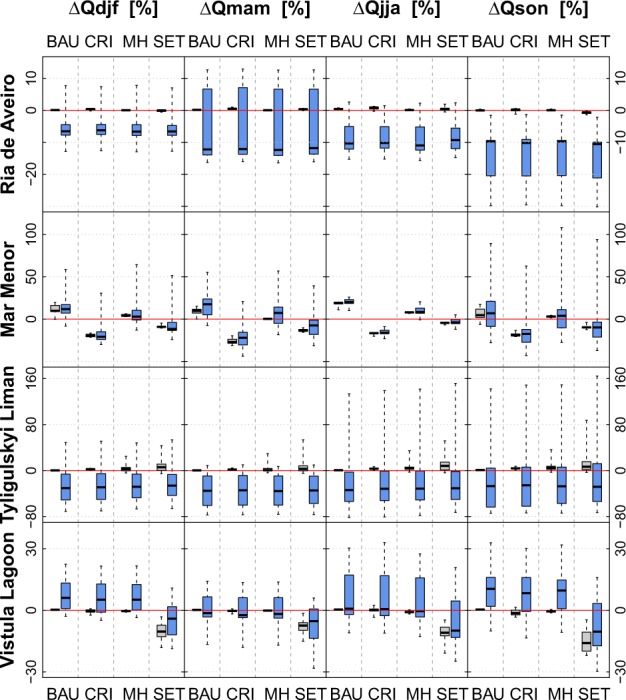
Long-term average annual changes in seasonal water flows (winter—Q_djf_, spring—Q_mam_, summer—Q_jja_, and fall—Q_son_) for each socio-economic scenario (BAU, CRI, MH, and SET) shown as boxplots. In gray, without the effect of climate change. In blue for the scenario period (2011–2040) compared with the reference period (1971–2000)

Figure 7 presents the impact assessment on seasonal discharge. There is very little variation on the impacts of the socio-economic scenarios on discharge throughout the year, similar to Q_av_.

In the *Ria de Aveiro* catchment most climate scenarios cause a decrease in winter (Q_djf_), summer (Q_jja_), and fall (Q_son_) flow (all scenarios), regardless the socio-economic scenario. Only, for spring flow (Q_mam_) an increase is simulated by some of them due to changes in the snowmelt processes.

In the case of *Mar Menor*, the BAU and MH scenarios have a higher impact on Q_jja_ compared with the rest of the year, as it is comprises mainly infiltrated irrigation water and released urban effluents. At the same time Q_jja_ is the one least influenced by climate change, whereas Q_son_, which is generated mostly natural, is very sensitive to climate change and shows the highest uncertainty among the four seasons.

Modeling results for the *Tyligulskyi Liman* catchment under the combined scenarios suggest an average decrease in discharge throughout the year, irrespective of the socio-economic scenario. They indicate the smallest uncertainty for Q_mam_ and the higher for Q_jja_ and Q_son_, which corresponds well to the observed uncertainty in precipitation trends for these months.

In the *Vistula Lagoon* catchment, most climate scenarios project an increase in Q_son_ and Q_djf_ for the BAU, CRI, and MH scenarios, and weaken the decreasing trend of the SET scenario. Only Q_mam_ slightly decreases on average due to changes in snowfall and snowmelt processes. The disagreement between model outputs is larger for Q_jja_ and Q_son_ than for Q_djf_ and Q_mam_, which is also the case for the disagreement between climate scenarios regarding seasonal trends in precipitation (compare with Fig. 3).

#### Changes in major water cycle components

**Fig. 8:**
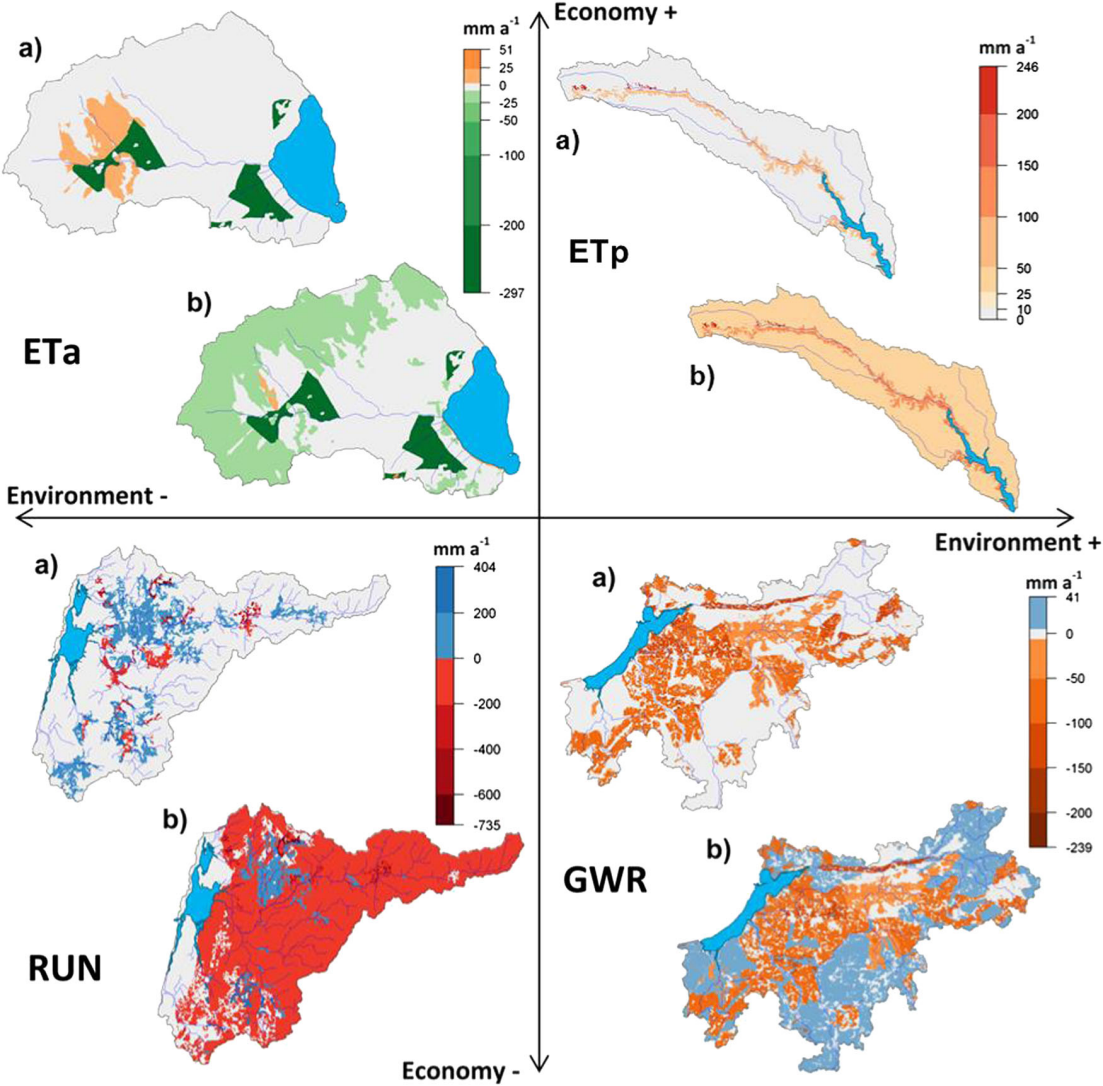
Example maps of long-term average annual spatial changes (mean of 15 climate scenarios in actual evapotranspiration (ETa) for the BAU scenario in the Mar Menor catchment, potential evapotranspiration (ETp) for the MH scenario in the Tyligulskyi Liman catchment, groundwater recharge (GWR) for the SET scenario in the Vistula Lagoon catchment and runoff (RUN) for the CRI scenario in the Ria de Aveiro catchment, showing **a** the socio-economic impacts only and **b** the combined impacts

The impacts on surface runoff (RUN), groundwater recharge (GWR), and actual and potential evapotranspiration (ETa and ETp) are summarized in Table 7. Examples of the spatial variability are presented in Fig. 8.

**Table 7:** Long-term average annual changes in runoff (RUN), groundwater recharge (GWR), actual evapotranspiration (ETa) and potential evapotranspiration (ETp) for each socio-economic scenario (BAU, CRI, MH, and SET) in the four study areas shown as combined and as socio-economic scenario impacts only (ses only)

		Ria de Aveiro				Mar Menor				Tyligulskyi Liman				Vistula Lagoon			
		Ses only		Combined		Ses only		Combined		Ses only		Combined		Ses only		Combined	
		mm a^−1^	%	mm a^−1^	%	mm a^−1^	%	mm a^−1^	%	mm a^−1^	%	mm a^−1^	%	mm a^−1^	%	mm a^−1^	%
BAU	RUN	1.33	0	−37.04	−6	0	0	0	0	0	0	−2.08	−15	0.61	1	9.90	12
	GWR	−0.19	0	−55.96	−6	−2.03	−9	−2.30	−2	0	0	−3.12	−16	0.61	1	10.23	12
	ETa	1.26	0	−5.49	−1	−22.95	−5	−24.41	−5	0	0	−10.45	−2	−0.89	0	8.96	2
	ETp	0.27	0	68.95	7	−4.05	0	26.10	1	0	0	40.67	5	−0.07	0	14.90	1
CRI	RUN	2.86	1	−35.52	−6	0	0	0	0	0.27	0	−1.81	−14	−4.87	−5	4.42	6
	GWR	1.00	0	−54.77	−6	−3.80	−16	−4.06	−10	0.29	0	−2.83	−15	−4.89	−5	4.73	6
	ETa	−2.22	−1	−8.98	−2	−46.25	−9	−47.70	−9	−0.64	0	−11.08	−2	2.05	0	11.90	2
	ETp	1.86	0	70.54	7	−4.71	0	25.44	1	−3.92	0	36.74	5	1.57	0	16.54	2
MH	RUN	−0.11	0	−38.49	−6	0	0	0	0	−1.27	−4	−3.35	−19	0.10	0	9.32	12
	GWR	−0.09	0	−55.86	−6	0.37	1	0.11	10	−1.33	−4	−4.45	−20	1.67	0	9.72	12
	ETa	0.67	0	−6.09	−2	6.16	1	4.70	1	3.13	1	−7.32	−2	−0.40	0	11.52	2
	ETp	−0.17	0	68.51	7	−3.19	0	26.97	1	7.71	1	48.38	6	0.10	0	14.57	1
SET	RUN	4.61	1	−33.76	−6	0	0	0	0	−3.78	−12	−5.86	−25	−24.40	−25	−15.22	−17
	GWR	0.01	0	−55.76	−6	−1.98	−9	−2.24	−1	−3.89	−12	−7.01	−26	−24.50	−25	−14.88	−17
	ETa	4.75	1	−2.00	0	−26.02	−5	−27.48	−5	5.57	1	−4.88	−1	27.96	5	37.81	7
	ETp	0.51	0	69.18	7	−4.05	0	26.10	1	3.06	0	43.73	6	5.00	1	19.96	2

In the case of *Ria de Aveiro*, the socio-economic scenarios have no significant impact on the major water cycle components on average, whereas the combined impacts correspond well to the climate change signals. GWR and RUN are projected to decrease by 6% in response to the decrease in average annual precipitation. In addition, the spatial pattern indicates some changes that are related to vegetation cover. For example, in the CRI scenario more runoff is generated on deforested areas, as these have lower transpiration rates, which in this specific case allows higher surface runoff. The conversion of agricultural land into grassland causes a decrease in runoff in other areas due to improved soil permeability and water infiltration rates (lower curve numbers) and an overall higher transpiration rate (grassland has a permanent vegetation cover). ETp is projected to increase, which is the result of both an increase in the amount of energy available to evaporate water (net radiation) and a decrease in the atmospheric moisture content (humidity).

In the *Mar Menor* catchment both the socio-economic and climate scenarios have only marginal impacts on RUN and ETp, as it is already extremely dry under reference climate. The reduction of the irrigated area in the BAU, CRI, and SET scenarios causes a clear decrease in GWR and ETa. In combination with climate change these trends are intensified. Moreover, ETa decreases in areas that are excluded from the irrigation zone but still cultivated, as less water is available for evapotranspiration, and increases in regions, where nonirrigated agricultural land is converted to fallow due to higher transpiration rates. These effects are mostly compensated by climate change.

In the *Tyligulskyi Liman* catchment, the BAU and CRI scenarios have no impact on the four water cycle components. The BAU scenario implies only a decrease in point sources and groundwater abstractions that have no influence on the water balance. In the CRI scenario half of the forested area is converted to fallow, and point sources as well as abstractions are reduced, but since forests account for <4% of the total area (Hesse et al. 2013), no significant changes are simulated. Only the decrease of ponds (MH and SET scenarios) seems to have a relevant effect, especially on RUN and GWR, although certain LUCs also have important implications. For example, a buffer zone along the Tyligul River that is implemented by converting agricultural land to grassland causes an increase in ETp and ETa. However, when looking at the entire catchment these changes are averaged out and the impacts become negligible. Climate projections intensify the decreasing trends in RUN and GWR by about 15%. This is five times higher than the average precipitation trend (3%) and shows nicely the vulnerability of the catchment toward climate change. Furthermore, climate change will likely lead to an increase of average ETp and a slight decrease of average ETa.

In the *Vistula Lagoon* catchment agricultural land is slightly increased in the BAU and MH scenarios, which has no significant impact on the water cycle components. The LUCs in the CRI and especially the SET scenarios on the other hand lead to a clear decrease in RUN and GWR. In both scenarios agricultural land is reduced and mainly converted to fallow, which has higher rates of plant transpiration causing a reduction of water available for GWR. Moreover, in the SET scenario some parts of the agricultural land are converted to forest, which has the highest ETa rate of all vegetation types and thus contributes to the decrease in GWR while causing an increase in average ETa. The applied management changes in all four socio-economic scenarios are irrelevant for the water cycle. Furthermore, the precipitation and temperature trends (4% and 1.1 °C) in the combined scenarios cause an increase in all four components. We conclude that climate change is likely to reverse the trends caused by the CRI scenario and weaken the impacts induced by the SET scenario.

### Impacts on Nutrients

#### Changes in major nutrient loads

**Fig. 9:**
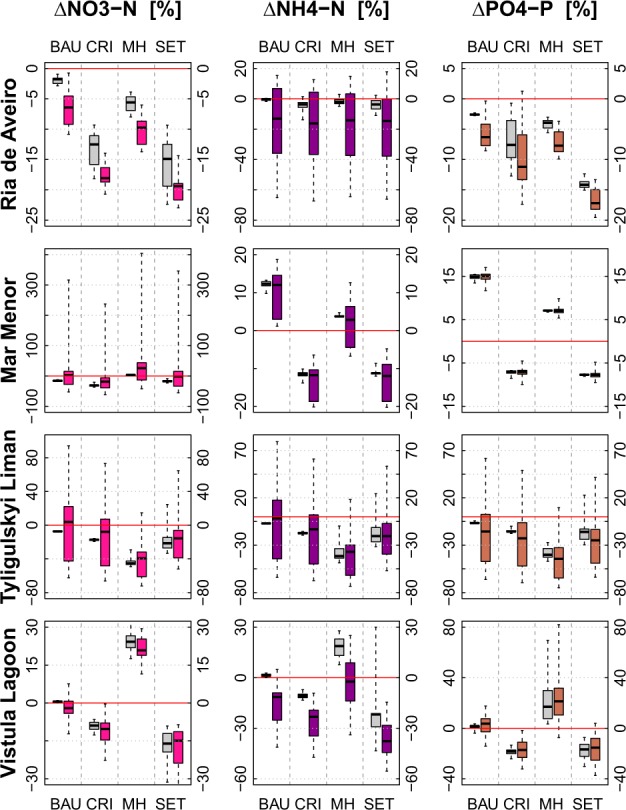
Long-term average annual changes in nutrient loads (NO_3_-N, NH_4_-N, and PH_4_-P) for four socio-economic scenario (BAU, CRI, MH, and SET) shown as boxplots with (in color) and without (in gray) climate change

Figure 9 summarizes the socio-economic and climate change impacts on nitrate nitrogen (NO_3_-N), ammonium nitrogen (NH_4_-N) and phosphate phosphorus (PO_4_-P) loads to the four lagoons.

The reductions of agricultural land and fertilizers cause a decrease of nutrient loads to the *Ria de Aveiro* (BAU, CRI, MH, and SET scenarios) and *Vistula Lagoon* (CRI and SET scenarios). In addition, the decrease of point sources assumed for all scenarios, except the CRI scenario in *Ria de Aveiro* also contributes to these trends. The observed impacts are further intensified under climate change, although the climate scenarios suggest a dryer climate for the *Ria de Aveiro* catchment only.

In the *Vistula Lagoon* catchment the decreasing trend in Q_mam_ (see “Changes in seasonal stream flow components”) causes this reduction, as fertilizers are applied mostly in April (Hesse et al. 2013). This fact also explains why climate change weakens the increase of nutrient loads to the lagoon in the BAU and MH scenarios. In both scenarios agricultural land is slightly increased and mineral fertilization remains unchanged (BAU) or is increased (MH), which leads to an increase of nutrient loads.

In the catchment of the *Tyligulskyi Liman* the fertilization rates are too low to significantly contribute to the overall water pollution, whereas the emissions from point sources strongly influence the ecological status of the catchment and the adjacent lagoon. In the MH scenario, even a drastic increase in mineral fertilization (500%) in combination with a reduction in point sources (50%) leads to a decrease in average nutrient loads by 40%. Lower rates of untreated waste water disposal also cause moderate to small decreases in nutrient loads in the other three scenarios. With regard to climate change, a clear interpretation is difficult, because the disagreement among climate scenarios is large and causes large ranges of uncertainty.

The effluents in the *Mar Menor* catchment are treated in a high-end UWWTP. Nevertheless, point source pollution is still an issue, especially during the peak season in summer. Moreover, intensive agriculture also contributes significantly to the overall nutrient input to the lagoon, and especially the NO_3_-N loads. For instance, under the BAU and MH scenarios the emissions from the UWWTP are increased, whereas the agricultural area (BAU scenario) and mineral fertilization rates (MH scenario) are reduced. These changes lead to higher NH_4_-N and PO_4_-P, and nearly constant NO_3_-N loads. Regarding climate change, PO_4_-P is not very sensitive, whereas NH_4_-N and NO_3_-N are moderately to strongly influenced by changes in the climatic conditions.

#### Changes in major transformation and transportation processes

**Fig. 10:**
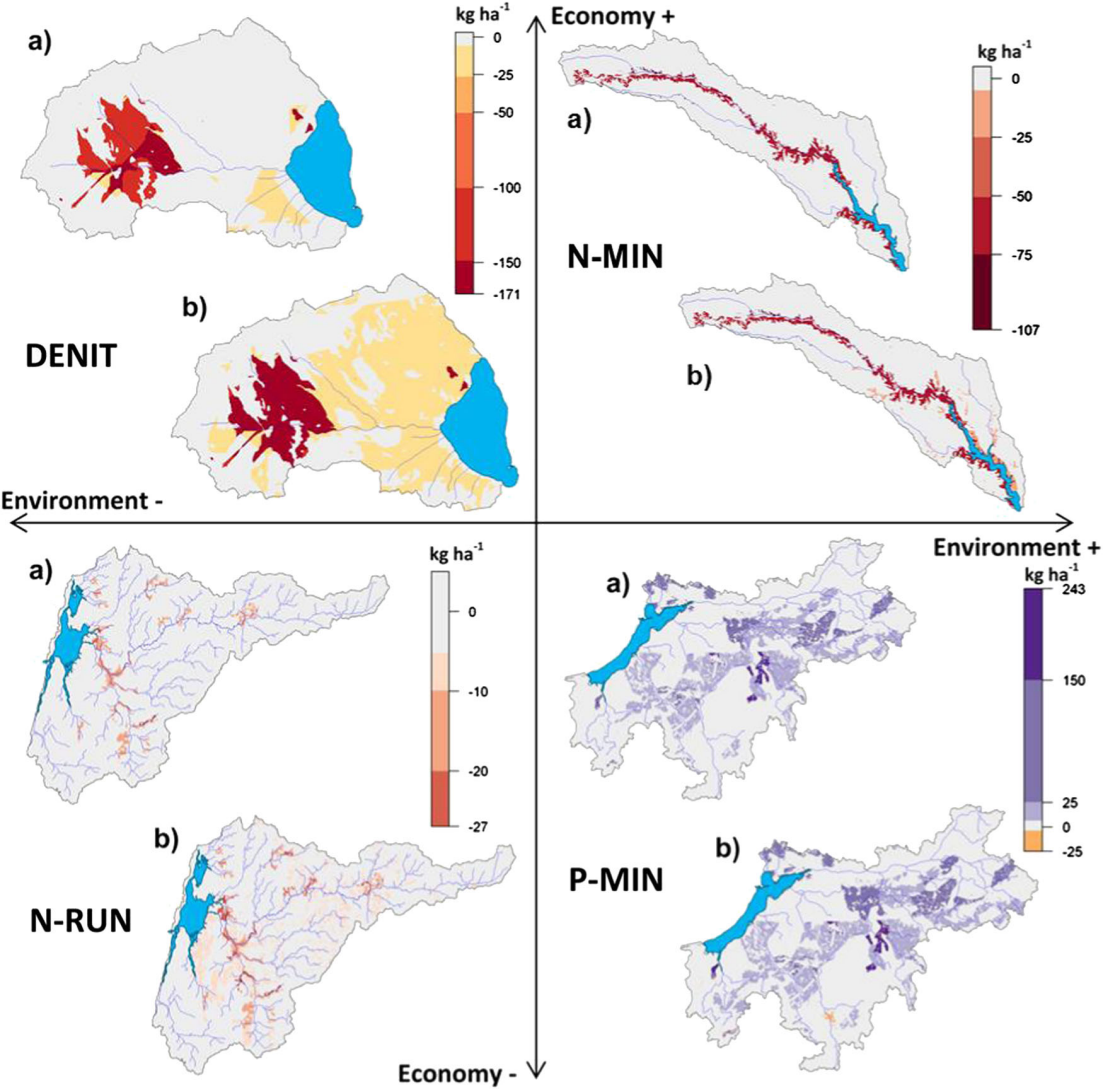
Example maps of long-term average annual spatial changes (mean of 15 climate scenarios) in denitrification (DENIT) for the BAU scenario in the Mar Menor catchment, nitrogen mineralization (N-MIN) for the MH scenario in the Tyligulskyi Liman catchment, nitrogen transported with runoff (N-RUN) for the CRI scenario in the Ria de Aveiro catchment and phosphorus mineralization (P-MIN) for the SET scenario in the Vistula Lagoon catchment showing **a** the socio-economic impacts only and the **b** the combined impacts

The simulated changes in major nutrient transformation and transportation processes are summarized in Table 8. Examples of the spatial variability of these processes are presented in Fig. 10.

**Table 8:** Long-term average annual changes in nitrogen transported with runoff (N-RUN), nitrogen mineralization (N-MIN), denitrification (DENIT), phosphorus transported with runoff (P-RUN) and phosphorus mineralization (P-MIN) for four socio-economic scenario (BAU, CRI, MH, and SET) in the four study areas shown as socio-economic (ses only) and combined impacts

		Ria de Aveiro				Mar Menor				Tyligulskyi Liman				Vistula Lagoon			
		Ses only		Combined		Ses only		Combined		Ses only		Combined		Ses only		Combined	
		kg ha^−1^	%	kg ha^−1^	%	kg ha^−1^	%	kg ha^−1^	%	kg ha^−1^	%	kg ha^−1^	%	kg ha^−1^	%	kg ha^−1^	%
**BAU**	N-RUN	0	0	−0.02	−14	−0.05	−17	−0.05	−17	0	0	0	0	0.09	3	−0.21	−6
	N-MIN	−1.75	−4	−1.99	−4	−4.50	−12	−3.15	−8	0	0	−3.58	−3	0	0	4.24	4
	DENIT	−0.79	−2	−1.45	−3	−19.03	−14	−20.26	−15	0	0	−0.80	−4	0.15	0	1.34	3
	P-RUN	0	0	0	0	0	0	0	0	0	0	0	0	0	0	0	0
	P-MIN	−0.18	−4	−0.29	−7	−0.34	−13	−0.33	−8	0	0	−1.59	−12	−0.13	−1	0.31	2
**CRI**	N-RUN	−0.01	−11	−0.03	−21	−0.09	−31	−0.09	−31	0	0	0	0	−0.17	−6	−0.46	−14
	N-MIN	−2.91	−6	−3.15	−6	−9.47	−25	−8.12	−21	0.59	0	−2.99	−2	−0.63	−1	3.62	4
	DENIT	−4.06	−9	−4.72	−10	−40.16	−29	−41.40	−30	−0.36	−1	−1.15	−5	−1.20	−3	−0.01	0
	P-RUN	0	0	0	0	0	0	0	0	0	0	0	0	−0.01	0	−0.01	0
	P-MIN	−0.24	−6	−0.35	−8	−0.70	−26	−0.69	−22	0.09	1	−1.50	−11	1.16	6	1.60	9
**MH**	N-RUN	0	0	−0.02	−14	0.01	3	0	0	0	0	0	0	1.06	36	0.76	24
	N−MIN	0.19	0	−0.05	0	1.34	3	2.69	8	−6.08	−5	−9.66	−7	8.72	9	12.97	13
	DENIT	−1.35	−3	−2.01	−4	6.03	4	4.80	4	2.71	9	1.91	5	7.78	17	8.97	20
	P-RUN	0	0	0	0	0	0	0	0	0	0	0	0	0.07	0	0.07	0
	P-MIN	−0.02	0	−0.13	−3	0.05	2	0.06	7	−0.48	−4	−2.07	−15	−0.65	−4	−0.20	−1
**SET**	N-RUN	−0.01	−15	−0.03	−24	−0.05	−18	−0.05	−18	0	0	0	0	−0.65	−22	−0.95	−29
	N-MIN	−5.14	−11	−5.38	−11	−5.42	−14	−4.06	−10	−11.19	−8	−14.77	−11	−5.73	−6	−1.48	−2
	DENIT	−4.91	−11	−5.57	−12	−22.13	−16	−23.37	−17	0.01	−1	−0.81	−4	1.47	2	2.66	5
	P-RUN	0	0	0	0	0	0	0	0	0	0	0	0	0	0	0	0
	P-MIN	−0.54	−13	−0.65	−15	−0.43	−17	−0.42	−12	−0.74	−6	−2.33	−16	5.50	29	5.94	32

The results are very diverse and different for each study area, with the exception of phosphate transported with surface and subsurface runoff (P-RUN), which shows no changes at all. In the case of *Ria de Aveiro* the reduction of fertilized agricultural land (BAU, CRI, and SET scenarios) and of N_min_-fertilizer (MH scenario) lead to lower denitrification rates (DENIT) in the catchment. The largest decrease is simulated for the CRI and SET scenarios, in which both agricultural land and N_min_-fertilizer are reduced. DENIT declines only slightly in the BAU and MH scenarios, as agricultural land is reduced or remains constant, while N_min_-fertilizer is increased or reduced, respectively.

Except for N-MIN in the MH scenario, the mineralization of nitrogen and phosphorus (N-MIN and P-MIN) decreases in all socio-economic scenarios. These impacts can be related to a reduction in fertilization due to lower fertilization rates in combination with a decrease in agricultural land.

For the MH scenario, where agricultural land remains unchanged, only small changes are simulated. The decrease in mineral fertilizers in combination with an increase in N_org_-fertilizer cause a slight reduction in DENIT and P-MIN, and a small increase in N-RUN.

The transformation processes (N-MIN, P-MIN, and DENIT), may be also influenced by climate change, for example when environmental constraints, such as soil water content approaching saturation, are reached less frequently under a dryer climate. However, the impacts of climate change are smaller than those of the socio-economic scenarios, except for N-RUN, where projected precipitation clearly intensifies the socio-economic.

The BAU, CRI, and SET scenarios in the *Mar Menor* catchment lead to a decrease in DENIT, N-MIN, P-MIN, and N-RUN. In all three scenarios, agricultural area is reduced or partly excluded from the irrigation zone. In addition, N_min_- and P-fertilizer are reduced in the CRI and SET scenarios. Changes in point sources have no impacts on the nutrient transformation processes or their transportation with runoff, as they are directly added to the stream flow. The most significant impacts are simulated for the CRI scenario, which predicts the biggest land use and management changes. The strongest decrease in DENIT is simulated on former irrigated land that is converted to fallow. The abandonment of nonirrigated agricultural land causes a smaller decrease, and agricultural land that is no longer irrigated shows the least impact.

Under the future dryer climate the observed trends in DENIT are intensified and even extended to nearly the entire catchment. The decreasing trends of N-MIN and P-MIN are slightly smaller for the combined scenarios, as the dryer and warmer climate favors nutrient mineralization.

Contrary to the other three scenarios, the irrigated agricultural land increases in the MH scenario, along with the use of N_org_-fertilizer at the cost of N_min_-and P-fertilizers. These changes lead to some increases of the nutrient transformation flows in the catchment, which are slightly intensified for N-Min and P-MIN and weakened for DENIT by climate change.

In the catchment of the *Tyligulskyi Liman*, only the SET and MH scenarios imply changes that are relevant for DENIT, N-MIN and P-MIN. N-MIN and P-MIN are projected to decrease for both scenarios by 4–8%. Figure 10 shows that N-MIN is drastically reduced inside the green corridor along the Tyligul River, where agricultural land has been converted to grassland. Removing fertilization in this area causes a strong decrease in the N_org_ and P_org_ pools, which leads to a decrease in N-MIN and P-MIN. The reduction of agricultural land also leads to a decrease, but the drastic increase of N_min_-fertilizer (500%) in the MH scenario causes an overall increase in DENIT. In the SET scenario 30% of agricultural land is abandoned and N_min_-fertilizer is increased by only 200%. Consequently, a stronger decrease in N-MIN and P-MIN than in the MH scenario and a slight decrease in DENIT are simulated.

Similar as in the other two catchments, a dryer climate is expected to intensify the observed decreasing trends and weaken the increasing trend of DENIT for the MH scenario in future.

In the case of *Vistula Lagoon* the abandoning of agricultural land has different impacts on some of the nutrient processes than in the other three catchments. In the SET scenario half of the agricultural land is converted to fallow and forest but N_min_-fertilizer and P-fertilizer are increased, which causes higher DENIT (+2%) and P-MIN (29%) as well as lower N-MIN and N-RUN (−6% and −22%). N-MIN and N-RUN decrease as the new land use types imply lower anthropogenic N input to the system, and lead to substantially lower runoff in the catchment. DENIT increases slightly, as it is less affected by LUC than but by an increase in N_min_-fertilizer. P-MIN is clearly higher on former agricultural areas that have been converted to fallow (see Fig. 10). This type of LUC leads to a decrease in P-MIN in the other three catchments. However, in the case of *Vistula Lagoon* there is additional input of P_org_ from plant residue under the new land use type that compensates for the absence of input through fertilization and even leads to an increase of the P_org_ pool and subsequently to an increase in P-MIN. This increase is 6% only in the CRI scenario, as the assumed LUCs are smaller. Accordingly, P-MIN is higher in the BAU and MH scenarios, as these two assume a slight increase in agricultural land. Moreover, in the MH scenario, N_min_-, P-, and N_org_-fertilization are also drastically increased (+100%, +100%, and +300%), which adds up to an increase of N-MIN (+9%), DENIT (+17%), and finally N-RUN (+36%) in the catchment.

A wetter climate in future suggests that soils will reach saturation more frequently, which may intensify the increasing trend in mineralization in the CRI and SET scenarios, weaken the decreasing trend in the MH scenario or even reverse it in the BAU scenario. However, more precipitation does not automatically mean a higher nitrogen input to the lagoon, as spring flow is projected to decrease on average, which may lead to a reduction of N-RUN.

## Summary

This paper addresses the issue of including potential trends of economic development and environmental awareness in climate impact research. The study aims to provide a sound scientific basis for the development of pan-European management recommendations for coastal areas. We therefore assessed and compared the effects of climate change on water availability and nutrient loads under various land use and management settings in the catchments of four European lagoons: *Ria de Aveiro*, *Mar Menor*, *Vistula Lagoon*, and *Tyligulskyi Liman*. Different setups of the eco-hydrological model SWIM, each representing one reference and four plausible socio-economic scenarios (the business as usual—BAU, crisis—CRI, managed horizons—MH, and set-aside—SET scenarios) for the four case study areas were driven by downscaled climate scenario data for a reference (1971–2000) and a near future scenario period (2011–2040).

The hydrological regimes in the *Ria de Aveiro* and *Vistula Lagoon* catchments are relatively natural, and the simulated impacts correspond well to the projected trends in precipitation. Looking at simulated changes averaged over climate scenarios, we can see that the analyzed discharge and water cycle components decrease (−5 to −15%) in the *Ria de Aveiro* and increase (5–10%) in the *Vistula Lagoon* catchment, more or less regardless of the applied socio-economic scenario. Only under the SET scenario for the *Vistula Lagoon* catchment, which represents a decreasing economic development and increasing environmental awareness water availability is reduced by the applied land use and management modifications, despite the increasing trend in precipitation for this region.

In the intensely managed *Mar Menor* catchment, the socio-economic scenarios determine the direction of changes, while climate seems not to have a significant influence on the hydrological regime. However, the impacts of a decrease in irrigated area that is assumed for three of the socio-economic scenarios can be indirectly related to climate change, or more specifically to a decrease of water availability in the donor basin of the Tagus-Segura IBWT (Kilsby et al. 2007; Lobanova et al. 2016; Morote et al. 2017).

In the most eastern case study area, the *Tyligulskyi Liman* catchment, water resources are strongly regulated and the applied socio-economic scenarios, except the one following current trends (BAU) lead to a little increase in water availability. However, this increase is mostly reversed under climate change, as precipitation is projected to decrease on average.

In the catchment of *Ria de Aveiro* all four socio-economic scenarios have a potential to intensify the decreasing trends in NO_3_-N and PO_4_-P loads due to climate change, while the impacts on the NH_4_-N loads are hardly influenced by the socio-economic scenarios but show a decrease on average under climate change.

In the *Vistula Lagoon* catchment, the socio-economic scenarios cause different trends and climate change has relatively little additional impact. The nutrient loads decrease on average under the BAU, CRI, and SET scenarios, and increase under the MH scenario, except for ammonium that shows no change under this scenario, despite the fact that climate scenarios project a wetter climate for this region.

In the *Mar Menor* catchment, the NO_3_-N loads are dominated by diffuse pollution from agricultural fields. Therefore they are highly vulnerable to both, climate and LUC, whereas the NH_4_-N and PO_4_-P loads respond mainly to changes in the quantity and composition of the released effluents.

And finally, in the *Tyligulskyi Liman* catchment, an overall decreasing trend in nutrient loads becomes visible when the four socio-economic scenarios are considered. It is also kept under most of the combined scenarios, despite the high disagreement among climate scenarios leading to a high uncertainty of projections.

## Discussion

The results show that the assessed impacts in the different lagoons have been diverse and difficult to “generalize”. For instance, looking at the socio-economic impacts only, this is a consequence of different initial conditions in the four catchments that lead to different and partly even contrasting quantifications of the same socio-economic scenario. When it comes to the combined impacts, the climate change signals for precipitation across Europe can be considered as the main cause. The overall trends detected in this study—a decrease in precipitation in the *Ria de Aveiro, Mar Menor* and *Tyligulskyi Liman* catchments, an increase in precipitation in the *Vistula Lagoon* catchment as well as specific seasonal trends—are also described in various other studies (European Environment Agency 2012; Graham 2004; Reihan et al. 2007; García-Ruiz et al. 2011; Giorgi and Lionello 2008) and lead inevitably to different trends in stream flow projections.

By combining the effects of climate change with potential socio-economic scenarios for the specific catchment, our study goes further than most of the climate impact assessments on water resources. However it still has some crucial limitations that are important to know with regard to the applicability of the results.

The climate scenarios used in our study are all based on the same emission scenario (A1B). We did not account for any of the other four SRES emission scenarios, neither for climate scenarios based on the more recent RCPs, which would be nice to do in a follow up study. Another scenario related limitation of this study are the applied socio-economic scenarios that represent four considerably clear future states of economic development and environmental awareness, but do not account for any intermediate possibility. Moreover the socio-economic scenarios are not applied in a dynamic way but are rather considered as a static state of the catchment in future, which certainly does not reflect reality and prevents the assessment of interim changes. They are also not physically based and, unlike climate scenarios we cannot evaluate their reliability nor carry out an uncertainty assessment. Nevertheless, the socio-economic scenarios are considered realistic. They were developed together with stakeholders, which increases the likelihood of our results to be actually considered by policy makers for decision-making. Finally the limited data availability, especially with regard to nutrient calibration and parameterization of ungauged streams has to be mentioned at this point, as it is another reason why the results of the impact assessment should be considered critically.

Nevertheless, this study provides suitable measures for each of the four study areas to successfully face climate change impacts. For the *Ria de Aveiro* lagoon, for example, deforestation in the central part of the catchment might have a positive effect (increase in total inflow) from a hydrological point of view. However, in case such a measure is considered the potential impacts for other important sectors, such as forestry, nature conservation, or tourism, to name only few, would have to be assessed first. Moreover, the effects of the increased inflow to the lagoon might not be necessarily beneficial for its salinity level, the hydrodynamics, the sediment loads, the shellfish breeding, or seaweed growth. In the case of the *Mar Menor* catchment, an expansion of the already existing irrigation zone could mitigate negative climate change impacts on the groundwater levels by adding additional water from outside the catchment (Tagus-Segura IBWT) to the system. The *Tyligulskyi Liman* could receive more freshwater from its catchment under the projected dryer future if the effective volume of operating ponds is at least halved. Moreover, similar as in the *Ria de Aveiro* catchment the groundwater resources could benefit if some forested area is converted to fallow. Apart from climate induced threats, the modeling suggests that a green corridor along the river could have a positive effect on the nutrient loadings to the lagoon (decrease nutrients), which might become important when future freshwater inflow decreases and nutrient concentrations increase. And finally, with regard to the *Vistula Lagoon* catchment, which seems not be threatened by climate change in the near future any reduction of point source and diffuse pollution is recommended, which of course is also the case for the other three catchments.

Some of the study outputs have been already used in follow up studies (e.g., lagoon modeling, Bielecka et al. 2015), within specific frameworks (e.g., Dolbeth et al. 2016) or could find further applications in the future. It is therefore important to acknowledge the key limitations and uncertainties of these.

## Conclusions

Our results show that the implications of potential socio-economic changes can intensify, weaken, or even reverse the effects of global warming. This underlines the need for impact studies to equally address both, climate change and socio-economic changes. Given the fact that the assessed impacts are very heterogeneous, which demonstrates the uniqueness and complexity of each catchment, we cannot formulate specific management recommendations at pan-European level, as initially intended. We would rather like to stress the importance of regional studies in the context of climate change adaptation or mitigation strategies for coastal areas.

## Supplementary information

Supplementary Fig. 1

Supplementary Fig. 2

Supplementary Fig. 3
