# Supplementary figures and images for: Assessment of Socio-Economic and Climate Change Impacts on Water Resources in Four European Lagoon Catchments
Source: Environ Manage. 2019 Nov 8;64(6):701–20. doi: 10.1007/s00267-019-01188-1 (PMC6875548; doi:10.1007/s00267-019-01188-1)

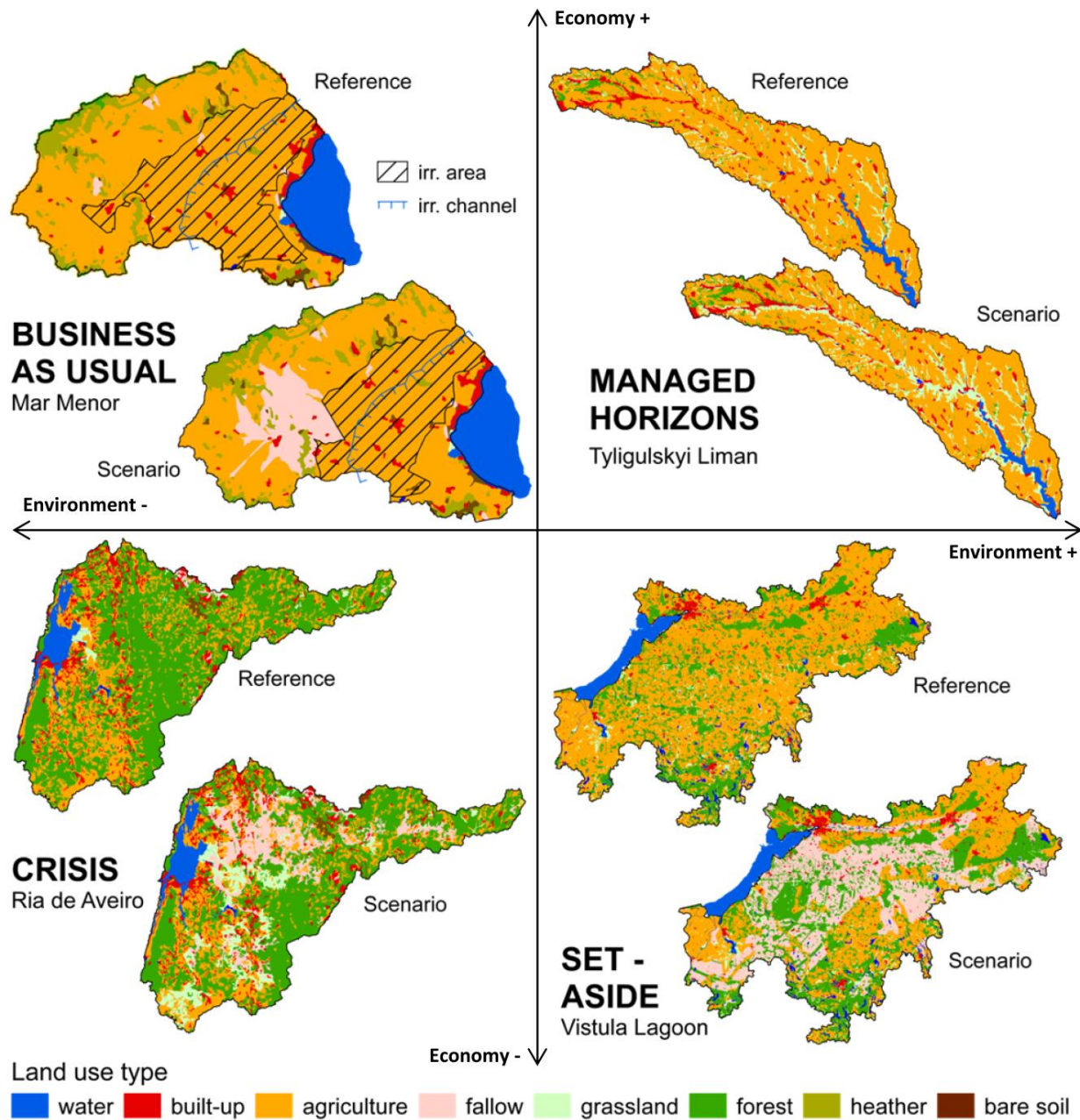

Supplement: Supplementary file 1 — Supplementary Fig. 1 [file 267_2019_1188_MOESM1_ESM.pdf]

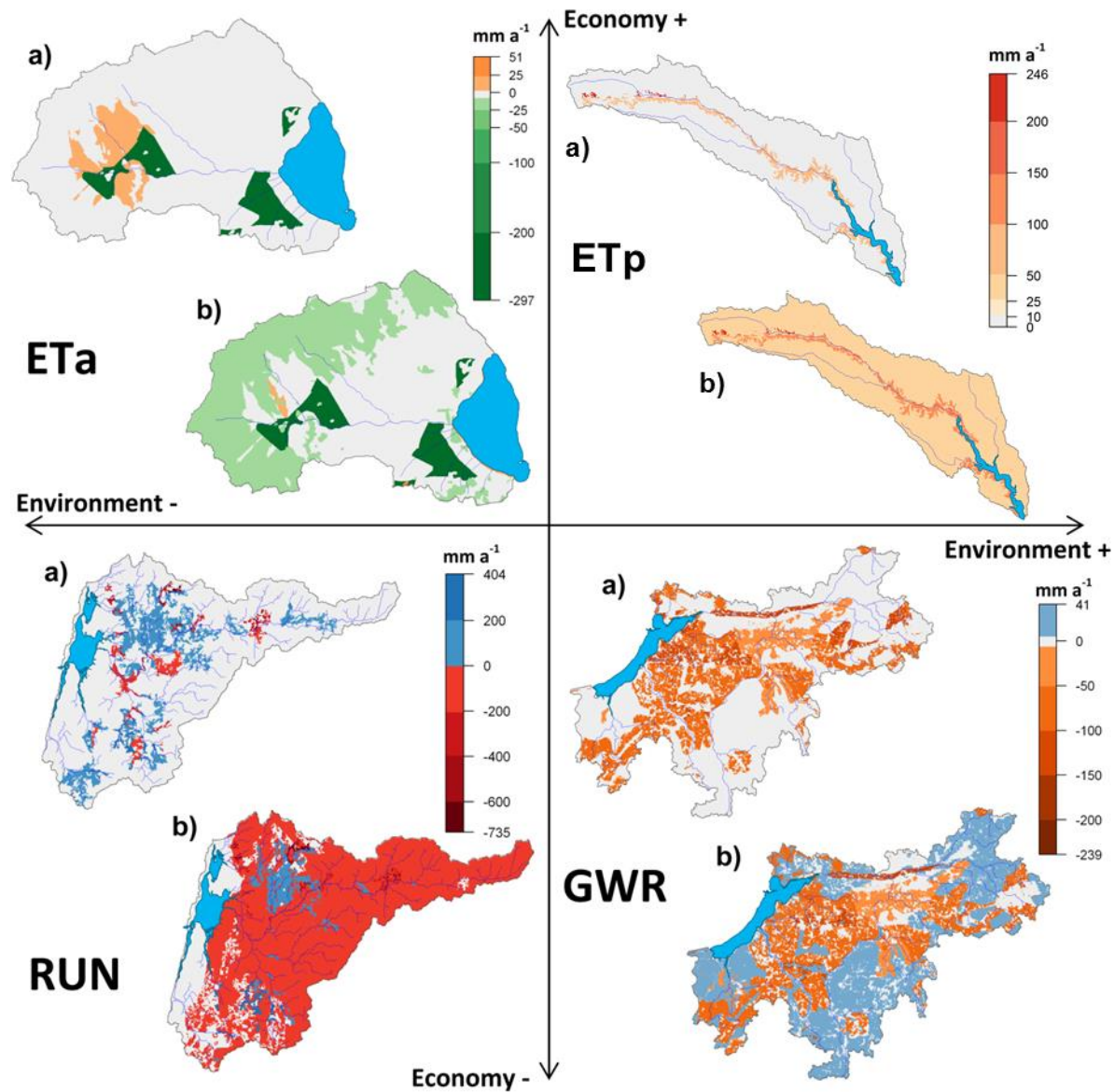

Supplement: Supplementary file 2 — Supplementary Fig. 2 [file 267_2019_1188_MOESM2_ESM.pdf]

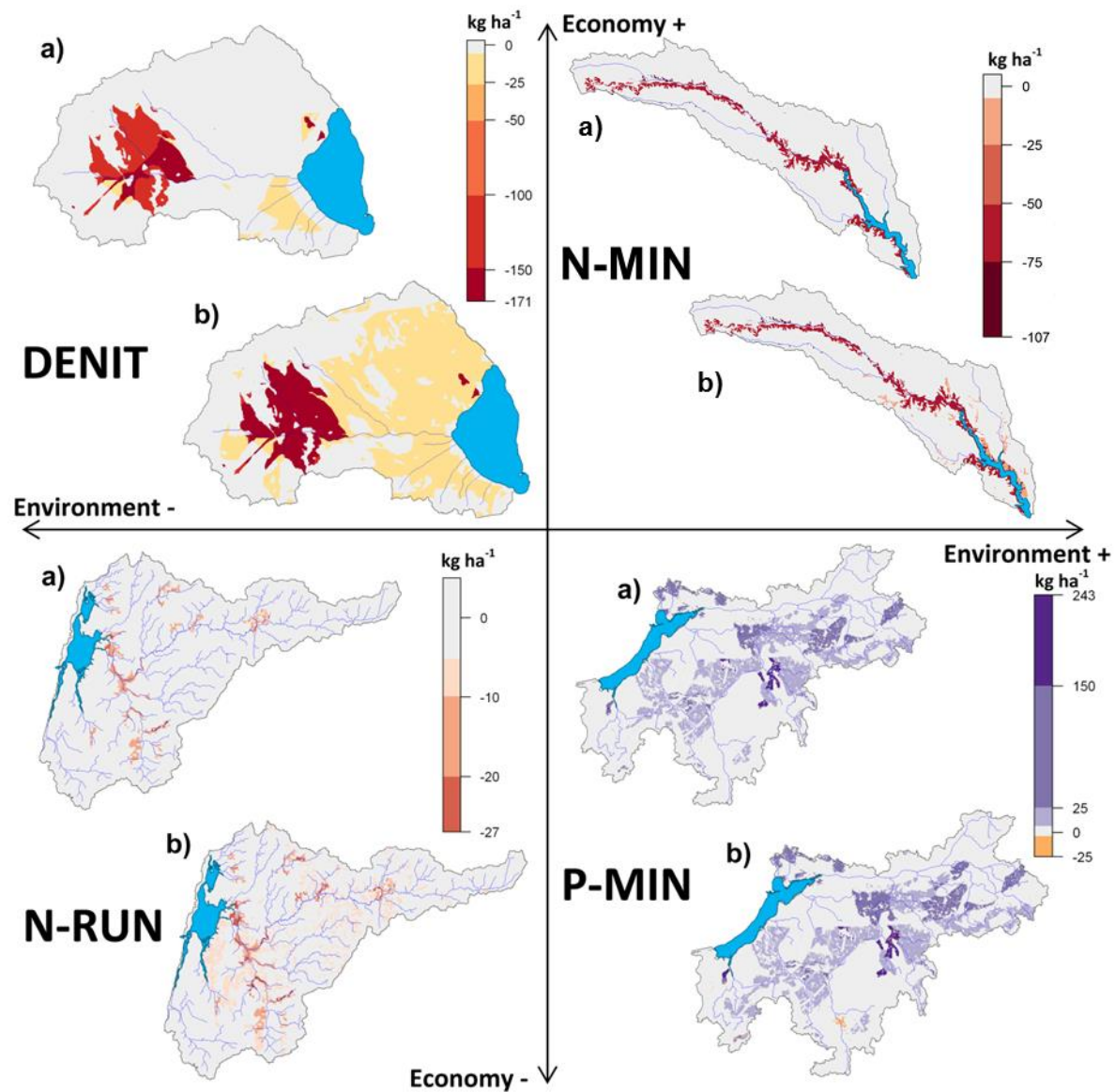

Supplement: Supplementary file 3 — Supplementary Fig. 3 [file 267_2019_1188_MOESM3_ESM.pdf]
